# Transitioning Between Online Gambling Modalities and Decrease in Total Gambling Activity, but No Indication of Increase in Problematic Online Gambling Intensity During the First Phase of the COVID-19 Outbreak in Sweden: A Time Series Forecast Study

**DOI:** 10.3389/fpubh.2020.554542

**Published:** 2020-09-29

**Authors:** Philip Lindner, David Forsström, Jakob Jonsson, Anne H. Berman, Per Carlbring

**Affiliations:** ^1^Department of Clinical Neuroscience, Centre for Psychiatry Research, Karolinska Institutet & Stockholm Health Care Services, Region Stockholm, Stockholm, Sweden; ^2^Department of Psychology, Stockholm University, Stockholm, Sweden; ^3^Department of Psychology, Uppsala University, Uppsala, Sweden

**Keywords:** gambling, pandemic (COVID-19), total consumption model, online gambling, time series

## Abstract

**Introduction:** The COVID-19 outbreak will likely have a public health impact beyond immediate disease transmission. Little is known about whether social distancing and other societal changes has provoked an increase in gambling, whether decreased betting opportunities due to paused sports events spurred gamblers to transition to online casino gambling, or whether any of these factors have had an impact on problem gambling.

**Methods:** Data on lookup queries against the Swedish Gambling Paus registry, logging all initiated gambling sessions by all licensed gambling providers, from 2019-01-01 (start of registry) to 2020-04-08 (well into the first phase of the outbreak) were analyzed using TBATS time series forecasting to estimate trends after the first domestic COVID-19 death. Obfuscated data on daily total wagered and deposited amounts, split by modality (casino or betting, and low and high intensity, respectively) for the equivalent period were supplied by a licensed online gambling provider.

**Results:** Total gambling activity decreased by 13.29% during the first phase of the outbreak compared to forecast. Analyses of online gambling data revealed that although betting decreased substantially in synchrony with a slight increase in online casino gambling, there was no increase in likely problematic, high-intensity gambling and neither did total online gambling increase.

**Conclusions:** This first, preliminary study revealed no increase in Swedish gambling activity, total or specifically online, in the first phase of the COVID-19 outbreak. Future research should examine whether pandemic-induced transitioning between gambling modalities and/or increased participation in gambling, leads to long-term effects on prevalence of problem gambling.

## Introduction

The ongoing COVID-19 pandemic (1) is estimated to have claimed over 600,000 lives, including over 5,600 lives in Sweden at time of writing in late July (2), and led to unprecedented global societal changes. With no pharmacological treatment or vaccine currently available, many countries have implemented regional or national quarantine procedures or encouraged other forms of social distancing strategies to curb the continued spread of the virus (3). Although burgeoning research supports the efficacy of social distancing strategies in combatting this pandemic (4–7), such measures are likely to also have a public mental health impact beyond the immediate effects on SARS-CoV-2 transmission (8).

In Sweden, public concerns were raised at an early stage of the outbreak that social distancing has increased gambling activity and possibly the prevalence of problem gambling (9), inciting the Swedish government to introduce temporary legislation (a decree, 2020:495) to the Swedish gambling market that includes a deposit limit on online casinos of 5,000 SEK per week (per gambling provider) and obligatory duration limits. Similar actions were taken in other countries with regards to gambling (10). The exact rationale and supporting evidence for this new Swedish legislation was not publicly disclosed and gambling providers reacted by raising the concern that these measures may disturb the delicate balance on the Swedish gambling market by channeling problem gamblers from licensed providers with responsible gambling obligations, to non-licensed providers lacking such obligations. At the time of this political decision, there was no published study on the effects of the COVID-19 outbreak on gambling. The extant literature did show that both anxiety (11) and boredom (12) are associated with problem gambling, both of which can be expected to increase during uncertain and threatening times (13) and in connection with social distancing procedures introduced to curb disease transmission (8). Previous research on the impact of economic recessions (burgeoning cases of which can already be seen following COVID-19 outbreaks) on gambling does however suggest a more complex association such that gambling may increase (particularly amongst those economically affected) but not necessarily gambling problems (14, 15). Moreover, specific to pandemics, the same social distancing phenomena that may increase some types of gambling likely have opposite effects on other types: pausing sport events means that there are fewer possibilities for betting, and closing physical casinos, bingo halls, and restaurants and clubs with slot machines, obviously reduces these types of gambling. Beyond investigating change in total gambling activity, studying such transition effects is another important research question since it is well-established that different gambling modalities are associated with different risks for developing gambling problems, both empirically (16, 17) and theoretically (18).

At time of writing, four studies have examined the impact of the COVID-19 outbreak on gambling: three cross-sectional survey studies (10, 19, 20) and one using aggregated time series from a multinational gambling provider (21). Two deserve special mention in the context of the current study. The time series study used behavior tracking data from gamblers in Sweden, Germany, Finland, and Norway (not taking different pandemic development courses into account) and found not only the expected substantial decrease in betting activity during the first phase of the COVID-19 pandemic, but also that the percentage of previously active betters who also played casino games decreased from pre-pandemic levels, i.e., no evidence of gamblers transitioning between modalities (21). A population survey study conducted in Sweden found that only 4% of gamblers reported gambling more during the COVID-19 crisis, 51% reported no difference and seven percent reported gambling less (the remaining percentage did not gamble), only a few percentages of whom transitioned to other gambling modalities. Gamblers who reported increased gambling, and those who transitioned to other modalities were, however, more likely to have gambling problems (10). Importantly, without longitudinal data, or reliable and applicable stability estimates, one cannot say that the proportion who reported an increase or decrease in gambling is higher than what would be naturally expected. Further, the so called total consumption model (applicable to many regulated commodities) predicts a strong correlation between total population consumption and prevalence of excessive and problematic consumption (22), and there is robust evidence supporting the applicability of the total consumption model to the gambling field (23). Thus, the trend in total gambling activity following a pandemic remains a meaningful indicator and more research is needed to examine trends at both population- and subgroup-level, and split by gambling modality.

In the current study, we used two unique datasets to examine whether the first phase of the COVID-19 outbreak in Sweden was associated with an increase in overall gambling activity on a population level, whether high-intensity online gamblers were particularly affected, and whether online gamblers transitioned between gambling modalities. First, we used lookup-query data from the Swedish Gambling Authority's account database to estimate change in total gambling activity during the first phase of the outbreak. Since determining trends require a meaningful reference, several comparison trends were calculated to provide a robust measure of relative change, including from advanced time series forecasting with sliding-window sensitivity analysis. Second, aggregated data on daily amounts wagered and deposited at a large, licensed online gambling provider, split by gambling modality and gambling-intensity (respectively), allowed us to examine change in gambling patterns amongst high-intensity vs. low-intensity online gamblers, as well the extent of gamblers transitioning between gambling modalities.

## Methods and Materials

### Ethics and Study Design

Since the current study uses only aggregated data sets and did not involve research on individually identified human participants (relying only on population aggregated data), this study falls outside the applicability of the Swedish Act concerning Ethical Review of Research Involving Humans, and no independent ethical review is thus required. One of the datasets is publicly available through a freedom of information request to the Swedish Gambling Authority, whilst the other was made available to the researchers on condition of anonymity to the public, and secrecy as extended by the Swedish Public Access to Information and Secrecy Act (SF 2009:400).

### Setting and Data

Recent survey research suggests that around 0.6% of the Swedish population aged 16−87 are problem gamblers according to the PGSI definition and scoring interval (24), that an additional 3.6% present low-to moderate risk gambling, that online gamblers are overrepresented amongst problem gamblers, and that the prevalence of last-year online gambling increased slightly from 2015 to 2018, from 18 to 21% (25). Since 2018, Sweden has had a regulated gambling market with licensed gambling providers (26). Since January 1st 2019, there is a formal Gambling Pause registry (www.spelpaus.se), hosted by the Swedish Gambling Authority, available for anyone to use to exclude themselves from any type of licensed gambling for 1, 3, or 6 months, or until further notice. As a prerequisite for a license, gambling providers are required to have gamblers identify themselves to initiate any kind of gambling session, both online and on-site. On initiation of a gambling session, a lookup query is sent to the Gambling Pause registry, which returns information on whether or not the given individual is currently in the registry; a positive lookup excludes the gambler from any gambling activity.

**Figure 1:**
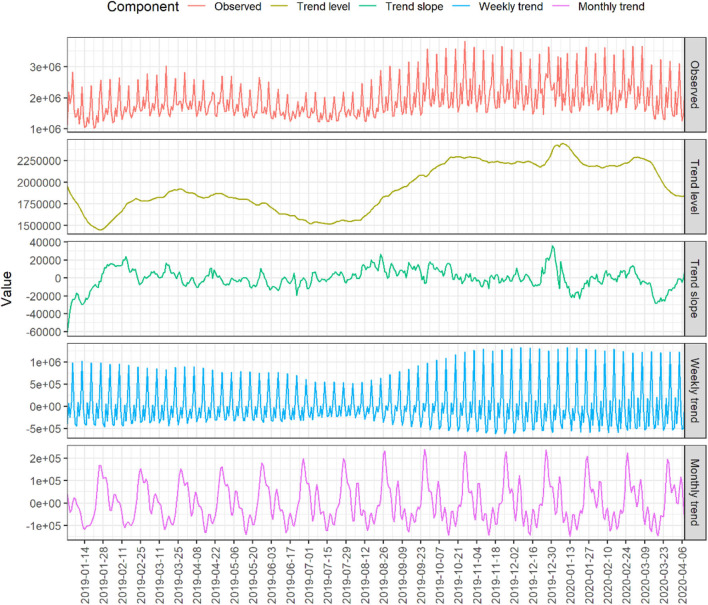
Observed time series and TBATS-derived components. For visualization purposes, Box-Cox transformation was suppressed (in order to preserve raw numbers) and trend included manually. Actual forecast models used AIC to select best specification. Displayed dates grouped into 2-weeks periods.

For the current study, the Swedish Gambling Authority provided data on number of logged lookup queries (i.e., attempted initiated gambling sessions) per day, from 2019-01-01 to 2020-04-08. For reasons unknown, the entry from 1 day (2019-06-10, well-before the original outbreak) was missing and replaced using trend interpolation from nearest neighbors, for a total of *k* = 464 entries. See Figure 1 for observed time series along with TBATS-derived components (27); see below for details. The data exhibited a strong weekly trend, with gambling activity at its highest on Saturdays, as in other time series on direct measures of gambling activity (21). The included monthly trend adequately captured the payday phenomenon (occurring on the 25th of each month, or closest weekday, for most working Swedes and those receiving any welfare benefits).

A second dataset covering the same period was provided to the researchers by one of the largest licensed gambling providers in Sweden. This dataset included four time series on daily total amount wagered or deposits, all obfuscated through division with a common, randomly selected number to preserve relative relationships between trends but rendering the absolute numbers non-meaningful. Data was split by two respective factors: total value of cash wagers in sports betting vs. online casino (traditional and live casino combined), and total value of deposits by high- vs. low-intensity players. There are many definitions of gambling intensity in the extant literature—in the current study, we used the same definition as in the new temporary gambling legislation introduced in Sweden following the COVID-19 outbreak, explicitly referred to within a responsible gambling context which as per the main legislation (2018:1138) aims to protect players from excessive gambling. For each individual player (account), deposits were aggregated on a (calendar) weekly level, and if the total deposited amounted exceeded 5,000 SEK (equivalent to roughly 480 Euro at time of writing), any deposited amount by that player that week was classified as high-intensity, otherwise as low-intensity. This aggregation was done by the gambling provider prior to data sharing. Of note, the limit of 5,000 SEK per week corresponds to roughly 83% of a Swedish median net income, and equals approximately twice the monthly limit mentioned in the Swedish Gambling Decree (2018:1475) for when a gambling provider is obligated to contact the gambler as a responsible gambling obligation.

### Analyses

All analyses were conducted using the R 3.6.3 statistical environment. Reproducible code, along with the lookup-query data, can be found at an online repository (28).

Lookup-query data was used to examine change in total gambling activity in the first phase of the COVID-19 outbreak in Sweden. The point of reference is pivotal in determining directionality of any trend. In the current study, we calculate several such references. For our primary analysis on change in total gambling activity, we used automated TBATS time series analysis (27), as implemented in the R forecast package (29), to model trends in gambling activity leading up to the COVID-19 outbreak. Box-Cox transformation, trend inclusion (with or without dampening) and use of ARMA errors (if so, with back-transformed means) was determined automatically by best fit according to AIC.

The Swedish government's response to the COVID-19 outbreak has (thus far) been characterized by an emphasis on voluntary social distancing measures, along with some structural measures like closing high schools and colleges (moving all teaching online), and prohibiting public gatherings of more than 500 and later 50 people. This entails that there is no obvious candidate date for when to begin forecasting (i.e., for establishing a trend to compare observed and forecasted values). See Table 1 for a timeline of events with a possible impact on registered gambling activity in Sweden; in brief, although the first COVID-19 case in Sweden was reported at the end of January, domestic disease transmission accelerated in early March and by mid-month, most major national and international sport leagues were paused. Although official domestic social distancing recommendations were issued during the second half of March, Google Mobility data (https://www.google.com/covid19/mobility/) suggests that voluntary social distancing had clearly begun before that. For the primary analysis on total gambling activity, we retained time series data up until 2020-03-11 (*k* = 436), the day of the first (highly publicized) death of a Swedish patient. Forecasts of the remaining *k* = 28 days were calculated using a TBATS model, and used as comparison references to determine trends in gambling activity following the COVID-19 outbreak. Mean percentage forecast error (difference between observed and forecast activity divided by forecast) during the period was calculated, with ordinary least-square regression used to calculate confidence intervals. In all but one analysis on forecast errors (including those described below), Durbin-Watson tests and ACF plots revealed no residual autocorrelation; in that case, a GLS model with AR1 correlation structure was used instead.

**Table 1:** Non-exclusive timeline of domestic and international events with a possible direct or indirect effect on gambling activity in Sweden.

**Date**	**Event**
2020-01-31	First COVID-19 case reported in Sweden. Public Health Agency requests COVID-19 be include in the Infectious Disease Act to allow legal action to curb the outbreak.
2020-03-06	First report of a COVID-19 case in Sweden requiring intensive care.
2020-03-09	Italian Serie A (popular betting event) pauses season.
2020-03-11	First Swedish casualty (made public same day). Public Health Agency recommends and government declares a limit on social gatherings to no more than 500 individuals, starting the next day.
2020-03-12	No audience allowed on-site during Swedish horse betting events, although racing and betting continues. American NHL pauses season.
2020-03-13	UK Premier League and German Bundesliga (popular betting events) to pause season.
2020-03-15	Swedish Hockey League (popular betting event) ends season prematurely.
2020-03-16	Public Health Agency recommends social distancing to individuals over 70 years old until further notice, and that people residing in Stockholm should work from home if possible.
2020-03-17	Public Health Agency recommends and government declares that secondary and tertiary education to be provided digitally across the country, starting the next day.
2020-03-24	Public Health Agency enacts regulation that restaurants, cafés and clubs (which may have slot machines) must take action to avoid crowding.
2020-03-27	Public Health Agency recommends and government declares a limit on social gatherings to no more than 50 individuals, starting the 2 days later.
2020-03-29	Swedish casinos close.
2020-04-01	Further advice issued by the Public Health Agency, including that everyone in the country keep a physical distance, that stores limit the number of simultaneous customers to avoid crowding, that employers allow employees to work from home if possible, and that risk groups avoid any social gathering.

To determine model performance and assess sensitivity of forecast period threshold date, the same TBAT model was re-run systematically, varying the window of entry inclusion from t minus 100 to *t* plus 14. Average percentage difference between observed and predicted values across the first 14 days of the forecast were calculated and compared to assess model performance. An average forecast discrepancy prior to the outbreak close to and normally distributed around zero, would signal good model performance. Should the difference grow in any direction when moving closer to a unknown latent turning point, and then reverse direction upon passing it, this would suggest that the initial forecast difference trend is driven by consequences of the outbreak (which can be expected to be linearly increasing), yet as more and more entries after the latent turning point are included in the model, the underlying trend will be better captured by the model and thus the forecast difference will again approach zero. Using the same forecast model, we also compared forecast discrepancies for the same last 2 weeks of the period regardless of window, expecting a similar overall pattern corresponding to the effect of period threshold date (i.e., sensitivity analysis). In addition to the forecast-derived reference, we also calculated three simpler, point-derived comparison references in the form of average activity in last 5, 10, or 25 same weekday prior to the outbreak, again with percentages against reference.

Data on daily deposited sums and wagers from the online gambling provider were used to examine differences in trends between high- and low-intensity gambling, and transitions between gambling modalities (betting and casino), respectively. First, TBATS models with the same primary forecast break of 2020-03-11 were run on each of the four time series and percentage forecast error calculated. Second, to provide comprehensible estimates of synchronization of trends in casino vs. betting activity, Loess time series decomposition (the mstl/msts functions in the forecast R package) with multiple seasonality (weeks and months) was used on each respective data set that included only the three preceding months (92 days) to isolate recent trends.

## Results

### Total Gambling Activity

**Figure 2:**
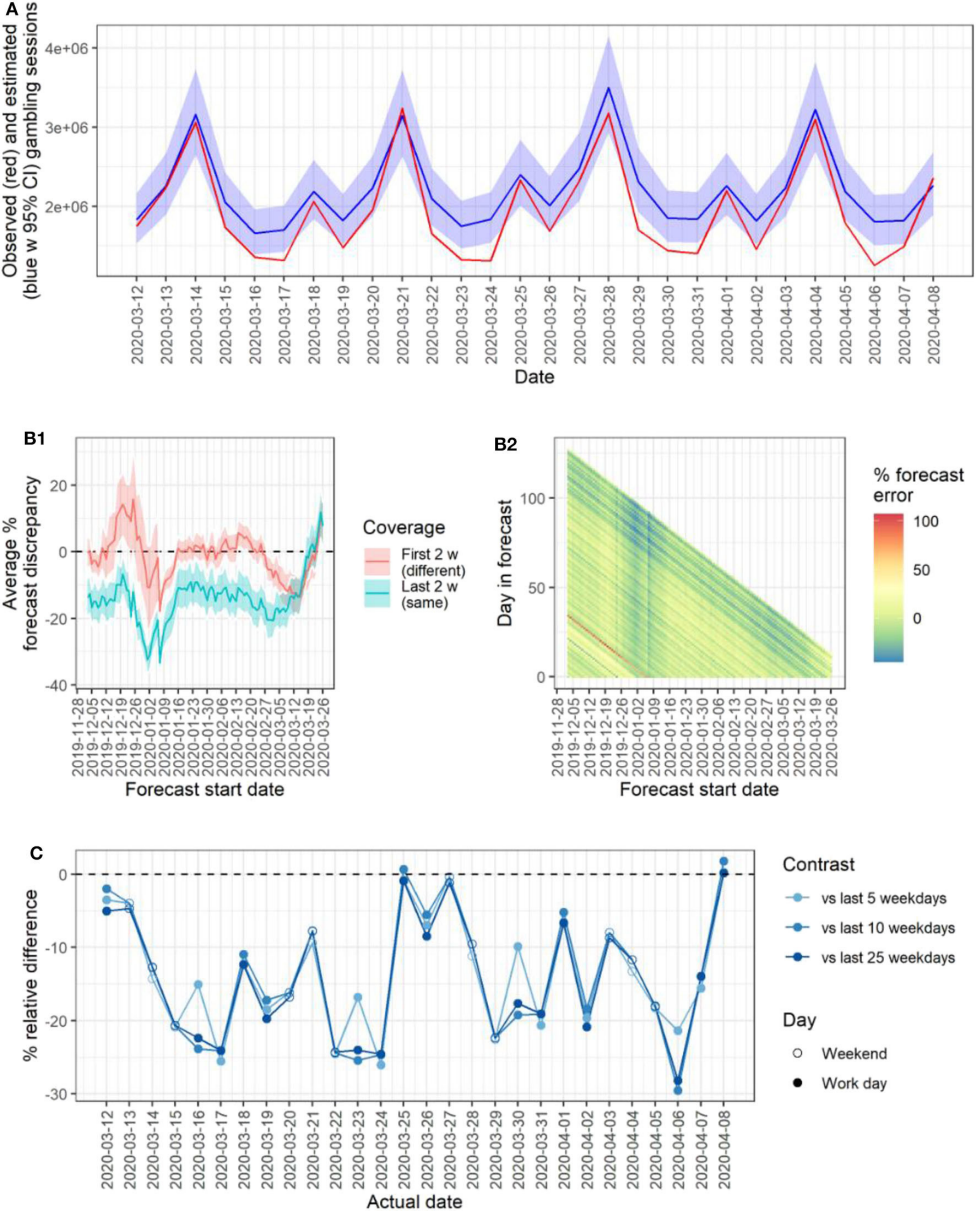
Observed gambling activity vs. time series forecast reference and point-estimate references. **(A)** Time series of observed (red) and forecasted (blue, with 95% CI) gambling activity during the evaluation period following the first COVID-19 death in Sweden. **(B1)** Expanding window analysis indicating forecast performance and sensitivity (respectively) by plotting period-average forecast discrepancy (dates group by week). **(B2)** Plot of forecast discrepancy for all dates and expanding window models (dates grouped by week). **(C)** Decrease relative to references points drawn from 5, 10, and 25 weeks (respectively) of same weekdays prior to 2020-03-12 (primary outbreak threshold).

Comparing observed gambling activity 2020-03-12 to 2020-04-08 to TBATS forecast calculated using data preceding this period, revealed a consistent and statistically significant overall decrease of 13.29% (95% CI: 9.37 to −17.21% decrease) compared to forecast. See panel A of Figure 2. Model performance and sensitivity analyses revealed the expected pattern when varying the window of included data: the average initial 2-weeks forecast discrepancy was typically small (<5%) and approximately normally distributed in the preceding 100 days, indicating good model performance. There was a distinct drop in performance around Christmas and New Year, resulting in discrepancies around ±20%; such a period effect is not unexpected since the model included no yearly seasonality, as there were not two full years of data. Importantly, good model performance returned and was stable for a period of several weeks prior to the outbreak. Sensitivity analysis revealed that the overall decrease in gambling activity remained negative until including data up till the 2020-03-20, after an accumulated period of almost 3 weeks of decreased gambling, as suggested by slope from full-model TBATS (Figure 1). See panel B1 of Figure 2 for period average forecast discrepancy in performance and sensitivity analyses, and panel B2 for all forecasted values across sliding window models.

Observed gambling activity post-outbreak was lower also when comparing to averaged point-estimates, with no obvious trend with increasing window of comparison. See panel C of Figure 2.

### Online Gambling Activity

**Figure 3:**
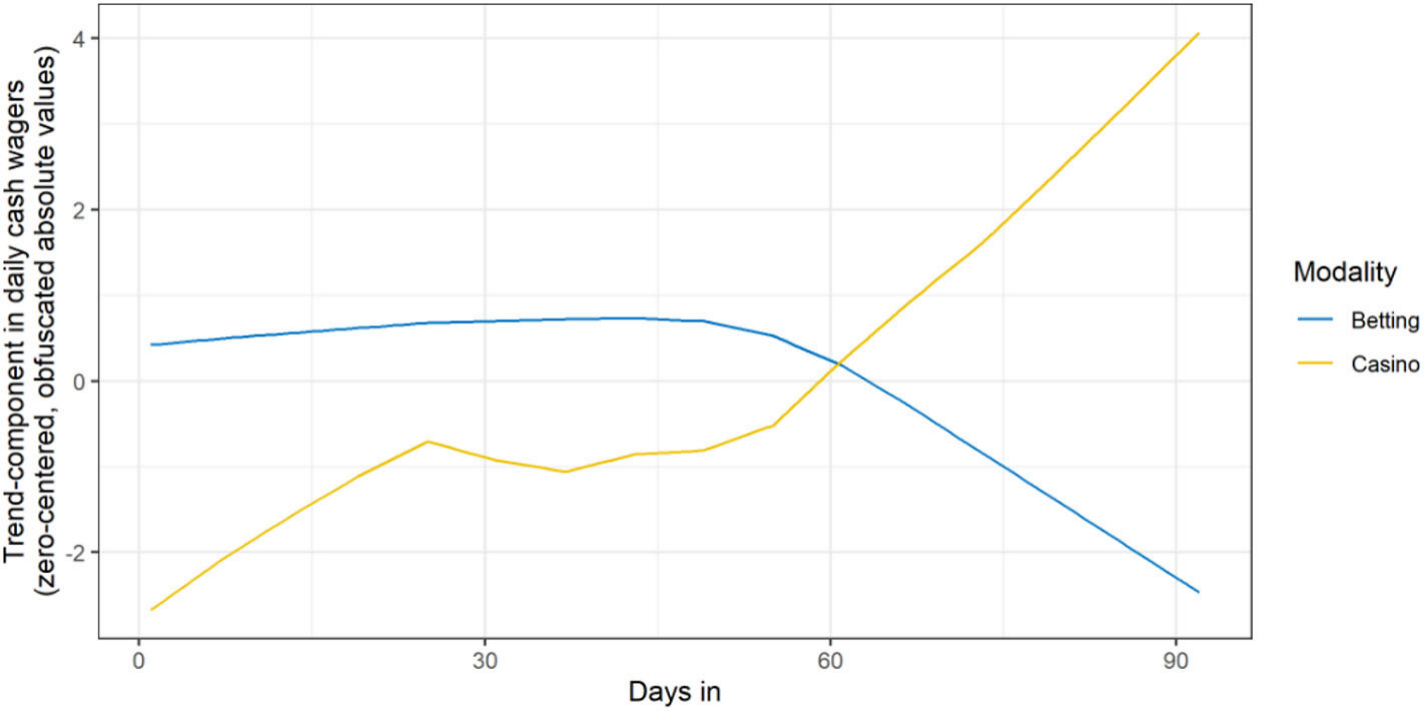
Trends in online betting and casino activity. For visualization purposes, each time series was zero-centered by subtracting their respective mean, preserving absolute differences.

Total online gambling activity after 2020-03-12 was 3.7% (95% CI:−2.7 to −10%) higher than the TBATS forecast, which was not significantly different from zero (*p* = 0.243). However, splitting online gambling activity by modality revealed a significant 74.8% decrease in betting (95% CI_AR1_:−84 to −65.6%) and a significant 8.63% (95% CI: 1.7 to −15.6%) increase in casino gambling. Plotting the decomposed trends revealed a synchronized increase and decrease for casino and betting activity respectively, beginning in late-February to early March, consistent with the trend in the lookup-query data and suggestive of transition effects. See Figure 3. However, TBATS modeling revealed no significant difference in low-intensity online gambling (95% CI:−7.9 to −5.2%, *p* = 0.678) compared to the TBATS forecast. High-intensity online gambling significantly decreased by 8.3% (95% CI:−14.3 to −2.3%, *p* = 0.009).

## Discussion

Using daily data on number of lookup queries against the national Swedish Gambling Pause registry—a high-quality measure of total licensed gambling activity—as well as data from an online gambling provider, the current study aimed to model trends in gambling during the first phase of the COVID-19 outbreak in Sweden. Comparisons against all references showed an overall decrease in total gambling activity in the range of 10–15%, estimated to have begun in early March, congruent with the sliding window model showing growing forecast discrepancy around this time. Data on online gambling showed the expected pattern of a substantial decrease in betting and slight increase in casino activity—synchronized in time—with total gambling showing no significant differences from forecast. There was no increase in low-intensity online gambling activity (as per the definition used in the temporary legislation introduced in Sweden) against forecast, yet a significant decrease in high-intensity gambling.

Our findings are not unexpected given that the examined total activity measure covers all types of licensed gambling, including sports betting, horse race betting, casino visits, and slot machine playing at restaurants and clubs, all of which have been affected to some degree by the social distancing strategies recommended or enforced by Swedish Authorities beginning in early-mid March (see Table 1 for timeline). Results are consistent with research showing a drop in overall gambling among adolescents in Norway following the banning of slot machines (30), a rapid supply reduction (31) similar to what was observed not only in Sweden but around the world as the COVID-19 pandemic began. Online casino gambling, the gambling modality most strongly associated with problem gambling (16, 17), remains naturally unaffected by the social distancing introduced to combat SARS-CoV-2 transmission, as does e.g. e-sports betting and to some degree horse race betting (which in Sweden continues but without an audience). To what extent online casino gambling would increase as betting and offline casino gambling decreased was unclear, with previous research suggesting no major transition effects (21) but that those that did transition were more likely to be problem gamblers (10).

Our combined findings suggest that although the expected transition between gambling modalities appears to have taken place, at least amongst users of a gambling provider that offers both online betting and casino games, this did not lead to an increase in problematic gambling as per the definition used in the new temporary Swedish legislation, or an increase in total online gambling indicative of increased problematic gambling. Further, under the assumption that the total consumption model is bi-directional, a decrease in total gambling activity should lead to a decrease of problem gambling, as cautiously supported by the current study (but see below). This is congruent with a survey finding that amongst Swedish gamblers who changed their gambling habits during the COVID-19 outbreak, it was twice as common to decrease rather than increase gambling; moreover, although problem gambling was relatively more common among those who gambled more, prevalence of problem gambling in absolute numbers was more than four times as common among those who did not gamble more (10). To what extent transitioning from online betting to casino playing, provoked by the COVID-19 situation, leads to long-term effects on prevalence of problem gambling (16–18) remains unknown and is an important topic for future research. Previous research shows that least amongst users of online gambling providers that offer both betting and casino games (as in the current study), there is likely to already be a great overlap such that a large proportion engage in both gambling types (21, 32), suggesting that most players who transitioned during the COVID-19 outbreak simply changed their proportion of betting vs. casino gambling. Importantly, our data does not allow us to distinguish between activity from transitions between gambling modalities amongst existing gamblers, from that of new gamblers who may have turned to gambling due to pandemic-related anxiety or boredom (11, 12). To what extent (new) gamblers that previously never wagered on online casinos, or gambled at all, begun doing so during the COVID-19 outbreak, should be investigated in future studies since this would arguably be a predictor of a long-term increase in population-level problem gambling. Whether online casino gambling decreases to pre-pandemic levels upon returning betting opportunities in the wake of lifted social distancing measures, will also be an important question for future research. Such research should also attempt to disentangle the unique effect of supply reduction in different types of offline gambling (e.g., physical casinos, restaurant slot machines).

An obvious limitation of the current study is that our proxy measure of problem gambling—high-intensity gambling, defined as depositing more than 5,000 SEK per week—rests on a high monetary threshold, equaling almost a net median Swedish income. Gambling habits may be problematic at much lower levels (33–36), which was also a prominent critique against the proposed legislation. Further, our population-level time series covered only a single metric (lookup-queries or wagered/deposited amounts). Future research using behavior tracking of individual accounts (37, 38) is needed to examine changes in gambling patterns in greater detail, also offering the possibility of examining subgroups of gamblers.

Some further limitations with regards to data and analysis should also be noted. First, while the official Gambling Pause registry provides high-quality data on total gambling activity, the fact that this registry has only existed since 2019-01-01 means that yearly trends cannot be reliably estimated, which could impact forecast performance and trend validity. However, expanding window analysis covering a period before the COVID-19 outbreak in Sweden, did suggest good forecast quality. Additionally, the data covers only gambling with licensed providers; recent survey findings however suggests that only 3% of gamblers gambled with an unlicensed provider last year (39). The Swedish Gambling Authority has raised concerns that the amount of login lookup-queries is higher than expected from estimates of gambling prevalence in Sweden, indicative of incorrect use of the API solution by the gambling providers. Although the Gambling Authority provides separate APIs for lookup-queries when the purpose is to check if someone is eligible for targeted advertisement, some gambling providers may incorrectly be using the login API also for this purpose, inflating the number of apparent logins and thereby total gambling activity. However, in order for this to be a confounding factor in the current study, the hypothetical percentage of incorrect API use would have to change in synchrony with the COVID-19 outbreak. Arguably, given the decrease in betting opportunities, it is more reasonable to expect an increase rather than decrease in advertisement activity during the COVID-19 outbreak, in which case the current study would have underestimated, not overestimated, the decrease in total gambling activity. Importantly, it should be noted that our two data sources revealed parsimonious trends, despite indexing different metrics and different populations, indicating robustness. Finally, the current study analyzed actual gambling data from a single online gambling provider that offers both casino games and betting, which likely increases the likelihood of users already being engaged in both types, decreasing the threshold for transitioning. Future research should therefore attempt to replicate these findings using independent datasets.

In conclusion, in contrast with publicly raised concerns (9), we found no indication of increased total gambling activity in the first phase of the COVID-19 outbreak in Sweden and the social distancing procedures introduced to combat it. Although betting decreased substantially in synchrony with a slight increase in online casino gambling, there was no increase in high-intensity, likely problematic gambling as per the Swedish government's own definition and neither did total online gambling increase. Future research is required to examine the impact of the outbreak over longer periods of time, on different types of gambling and on subgroups of gamblers, including problem gamblers, preferably using behavior tracking of individual gambling accounts.

## Data Availability Statement

One of the datasets is available at the referenced repository, and is also publicly available through a freedom of information request to the Swedish Gambling Authority. The other was made available to the researchers on condition of anonymity to the public, and secrecy (i.e. not publicly available), as extended by the Swedish Public Access to Information and Secrecy Act (SF 2009:400).

## Author Contributions

PL obtained data, performed analyses, and drafted manuscript. DF, JJ, AB, and PC made substantial contribution to the design of the study, analysis, interpretation of findings, and revised the manuscript for important intellectual content. All authors contributed to the article and approved the submitted version.

## Conflict of Interest

PL was supported by grants from the Centre for Psychiatry Research (Karolinska Institutet and Region Stockholm) and the Independent Research Council of Svenska Spel (a licensed, government-run gambling provider which has no influence on decisions by the independent council). DF was funded by the Independent Research Council of Svenska Spel, and the University of Bergen. JJ is an employee at Sustainable Interaction, a private company that provides responsible gambling services to the gambling industry. AB is a member of the Research Council of Svenska Spel and was supported by grants from Swedish Research Council (K2012-61-P-22131-01-6) and the Swedish Research Council for Health, Working Life and Welfare (2016-07091). PC reports having received funding for gambling related research from the Public Health Agency of Sweden, the Swedish Research Council for Health, Working Life and Welfare, the Independent Research Council of Svenska Spel, and the licensed gambling provider Ålands Penningautomatförening (Paf). JJ, AB, and PC have served as gambling experts for the Swedish National Board of Health and Welfare (Socialstyrelsen). PL, DF, JJ, and PC report ongoing and/or past industry-academia research collaborations on independent evaluations of responsible gambling tools. No funder or data provider had any role in the current research, which was conducted in the absence of any commercial interest.
